# Severe mental illness and the perimenopause

**DOI:** 10.1192/bjb.2023.89

**Published:** 2024-12

**Authors:** Sophie Behrman, Clair Crockett

**Affiliations:** 1 Oxford Health NHS Foundation Trust, UK; 2 Newson Health Menopause and Wellbeing Centre, UK

**Keywords:** Comorbidity, neuroendocrinology, organic syndromes, primary care, patients

## Abstract

Hormonal fluctuations in the perimenopause are associated with an array of physical and psychological symptoms. Those with pre-existing mental disorders may experience changes to their symptoms and response to treatment during the perimenopausal and postmenopausal periods and may also be at risk of poorer longer-term physical health outcomes in menopause. The transition towards menopause may be compounded by the oestradiol-suppressing effect of many psychotropics on the hypothalamopituitary–gonadal axis. A collaborative approach between primary care and secondary mental health services is an opportunity for proactive discussion of symptoms and support with management of the perimenopause. This may involve lifestyle measures and/or hormone replacement therapy, which can both lead to improvements in well-being and mental and physical health.

Perimenopausal hormone fluctuations lead to an idiosyncratic array of physical and psychological symptoms, which may lead to new-onset mental disorders as well as affecting pre-existing conditions. Patients with severe mental illness going through the perimenopause may expect changes in their symptomatology and response to treatment. Acknowledging and addressing perimenopausal symptoms with lifestyle measures and/or hormone replacement therapy (HRT) alongside relevant psychotropic medication leads to better well-being and physical and mental health outcomes for patients with severe mental illness. Owing to the complexity of patients with severe mental illness and the risk of diagnostic overshadowing, clinicians need to be proactive in asking patients about menopausal symptoms and inviting collaboration on management between secondary mental healthcare and general practice.

## Method

The authors independently sourced relevant studies using search terms including menopause*, perimenopause*, HRT, depression, anxiety, schizo*, psycho*, ‘bipolar affective’, hyperprolactin* in various combinations using PubMed, Google Scholar and ReadCube Papers in January and February 2023, and abstracts were screened for relevance to the review. Only peer-reviewed articles were accepted, and conference abstracts were excluded. References for background information (e.g. relevant guidelines) were specifically included in line with the authors’ knowledge of the field.

## Perimenopause, menopause and the brain

The menopause is when menstruation ceases owing to a reduction in hormone production from the ovaries. It is an indication that the ovaries are failing and no longer producing the hormones they have done throughout the reproductive years. The perimenopause represents a variable timeframe before the menopause when the hormones that are produced by the ovaries (oestradiol, progesterone and testosterone) can be highly fluctuant and responsible for many physical and psychological symptoms.

The average age of the menopause in the UK is 51, and perimenopausal symptoms are commonly seen during the transition towards this in the mid-to-late 40s, although they can occur much earlier. Patients may also experience an iatrogenic menopause, where ovaries may be surgically removed or their function affected by medical treatment such as chemotherapy, leading to a lack of hormone production.

The psychological symptoms that could be attributed to a typical menopause include anxiety, low mood, paranoia, anhedonia, irritability, dissociation, insomnia and feelings of low self-worth. These symptoms come about because of the impact of hormonal changes on the brain. Psychological symptoms associated with menopause may be sufficiently severe to reach ‘caseness’ for a mental disorder or may exacerbate underlying pre-existing mental disorders.

Oestradiol, progesterone and testosterone are important hormones in brain function, oestradiol most notably so. Oestradiol has a role in the modulation of serotonin, and serotonergic pathways are thought to be involved in the neurobiological basis of depression.^1^ When oestradiol levels fluctuate during the perimenopause and menopause, destabilising effects on mood can therefore occur.^2^ Other neurotransmitter pathways, including dehydroepiandrosterone sulfate and gamma-aminobutyric acid (GABA),^1^ are also affected by the menopause and thought to be linked to changes in mood.

In addition to oestradiol, total and free testosterone levels decline with age, often beginning in the mid reproductive years. There is evidence that transdermal testosterone therapy gives a meaningful improvement in psychological well-being^3^ as well as in the treatment of hypoactive sexual desire disorder.^4^

Progesterone receptors are also found within the brain, and reduced levels of progesterone, although not seen to have a consistent impact on psychological well-being, can have an impact on menopausal symptomology which would otherwise lead to an increase in psychological distress. An example of this is progesterone replacement improving insomnia; progesterone is converted to allopregnanolone in the brain, which leads to downstream GABAergic effects resulting in improvement in sleep.^5^ Improved sleep can in turn enhance emotional resilience.

## Pre-existing severe mental illness and the perimenopause

Fluctuations of hormone levels and the eventual decline in oestrogen levels during the perimenopause represent a risk factor for new-onset psychological symptoms (including anxiety, low mood and brain fog, which are commonly experienced in the normal course of menopause,^6^ and first episodes of mental illness, including depressive episodes^7^ and psychotic episodes^8^ among others).

Oestradiol is a potent neurosteroid and tends to be ‘neuroprotective’:^9^ stages of life where oestradiol is relatively low (premenstrually, postpartum, peri- and postmenopause) are associated with increases in depressive^10^ and psychotic^11^ episodes in women.

In this paper, we largely consider what happens to patients with pre-existing chronic mental illness who may be at risk of a relapse in symptoms^12^ or develop a need for higher doses of medication or new-onset treatment resistance.^13^ It is worth noting that some patients with mental illness seem to have a particularly hormonal signature to their relapse pattern (e.g. episodes of illness at hormonal events such as menarche, and premenstrually, perinatally and with some forms of hormonal contraception). This group of patients in particular are at risk of a decline in their mental health with the perimenopause.^1,14^

### Schizophrenia

Patients with schizophrenia are at risk of an exacerbation of their psychotic symptoms,^15^ perhaps due to declining oestradiol levels affecting neurotransmission in the brain and a loss of the ‘neuroprotectant’ effect of oestradiol.^9^ The reduction in oestradiol also has an impact on hepatic metabolism of antipsychotics, as some key liver enzymes are oestradiol dependent; this can lead to more effective metabolism of oral medication, leading to a reduction in efficacy for the same dose.^15^ This may go some way towards explaining why patients often experience increasing resistance to antipsychotic therapy over the course of their menopause.^13^

### Depression

Patients with a pre-existing depressive disorder are at risk of relapse in the perimenopausal period^12^ and may find that antidepressants which had previously helped are no longer effective,^12^ or that a much higher dose is required to have the same effect.^16^ There may also be a slightly different quality to the symptoms compared with premenopausal depressive episodes, the nuance of which is distinguished by the MENO-D questionnaire (Box 1)^17^ and summarised in Table 1.

Box 1 Prompt questions for MENO-D.

See www.maprc.org.au/sites/www.maprc.org.au/files/MENO-D_0.pdf for question responses and scoring details.

1. Over the last 2 weeks have you noticed reduced energy levels?
2. Over the last 2 weeks have you experienced increased paranoid thinking?
3. Over the last 2 weeks have you felt more irritable?
4. Over the last 2 weeks has your self-esteem been lowered?
5. Over the last 2 weeks have you withdrawn socially?
6. Over the last 2 weeks have you experienced heightened levels of anxiety?
7. Over the last 2 weeks have you experienced physical symptoms?
8. Over the last 2 weeks have you experienced sleep disturbance?
9. Have you gained weight (in comparison to pre-menopause weight)?
10. Over the past 2 weeks have you experienced a decreased libido?
11. Over the last 2 weeks have you noticed any memory related difficulties?
12. Over the past 2 weeks have you experienced problems concentrating?

**Table 1:** Comparison of presentation of perimenopausal depression with depressive episodes not associated with menopause

Symptom group	Presentation in perimenopausal depressive episode	Presentation in non-menopause-related depressive episodes
Mood	Low (but may fluctuate – ‘on/off’ phenomenon^12^)	Persistently low. May be some diurnal variation
Self-esteem	Extremely low	Low
Anxiety symptoms	Likely to be present	May be present
Negative cognitions (guilt, worthlessness)	May be present	Likely to be present
Sleep	Very poor. May be worsened by vasomotor symptoms	Poor. Early-morning waking.
Psychomotor symptoms	Retardation less common. May be some agitation associated with irritability	Agitation/retardation seen
Anhedonia	May be present	Often present
Weight	Increases	Decreases
Memory/concentration problems	Very common, especially word-finding difficulties	May be present
Libido	Low	Low
Paranoid ideation	Common	May be present
Aggression/rage	Common	Unlikely to be present
Dissociation	Common	May be present
Pain	Joint pains/muscle aches/headaches common	May be present

In England and Wales, the highest rates of suicide in women are seen in the 45–49-year-old age group (7.8 per 100 000 in 2021).^18^ Similar trends are seen in data from Australia; this has been examined and may be related to symptoms associated with perimenopause.^19^ A recent review by the Healthcare Safety Investigation Branch in response to a death by suicide of a perimenopausal woman has identified the management of perimenopausal symptoms in mental health services as a particular concern and tasked the Royal College of Psychiatrists with improving this.^20^

### Bipolar affective disorder

There is limited evidence on the course of bipolar affective disorder through the menopause, but preliminary evidence suggests that there may be an increase in the rate of mood disturbance, with a tendency towards depressive episodes^21^ and increases in irritability, hypomanic/manic symptoms and increased cycling also reported in case reports.^22^ It is worth considering the effect of perimenopause-associated insomnia as a mediating factor for this population, as those with bipolar are particularly sensitive to sleep disruption,^23^ although the mechanism has not been specifically examined.

Those who have had episodes of affective disturbance associated with childbirth are at particular risk of relapse in the perimenopause, particularly if this is an abrupt, iatrogenic menopause.^24^

### Anxiety

Anxiety symptoms are often a hallmark of the perimenopause and are more likely to occur in those with a pre-existing diagnosis or sensitivity towards anxiety.^25^ Although symptoms of anxiety (e.g. feeling tense) in menopause have been widely acknowledged and examined,^26^ research on specific anxiety disorders has been neglected.^27^ Anxiety symptoms are strongly linked to vasomotor symptoms (hot flushes), with anxiety symptoms preceding the onset of vasomotor symptoms.^28^

### Trauma

Adversity during early life is associated with a variety of subsequent mental disorders^29^ and also has an impact on the reproductive system, with an association with earlier menarche and potentially earlier menopause and a preponderance of vasomotor symptoms.^30^ Oestrogen may play a part in the stress vulnerability model to modulate the downstream effects of childhood trauma, including physical and psychological sequelae throughout the lifespan.^9^

### Neurodiversity

There is limited research specifically into the course of neurodiversity through menopause. People with autism spectrum disorders may find that the challenge of navigating a neurotypical world becomes enhanced at menopause, with increased sensory sensitivities and more communication and emotional regulation challenges, sometimes leading to new diagnoses being made around the perimenopausal period.^31^

Executive dysfunction and other cognitive problems are commonly reported during the perimenopause; these which can appear very similar to the constellation of symptoms seen in attention-deficit hyperactivity disorder (ADHD)^32^ and, like ADHD, can respond to stimulants.^33^ It is unclear whether this is an unmasking of underlying pre-existing ADHD or entirely *de novo* symptoms. Given the overlap, it can be hypothesised that those with pre-existing ADHD may experience a worsening of their ADHD symptoms with perimenopause, and it has been shown that people with comorbid ADHD are also more vulnerable to perimenopause-associated psychological symptoms.^34^

## Physical health risks associated with menopause

As levels of oestradiol, progesterone and testosterone decline during the perimenopause and into the menopause, we see an increase in a number of health risks. Postmenopausal patients are at increased risk of osteoporosis, cardiovascular disease, diabetes and cognitive decline. This is before mentioning the impact of perimenopausal and menopausal symptoms that can lead to a decline in overall well-being and difficulty in maintaining a healthy lifestyle, which both indirectly contribute to poorer physical health.^35^

## Physical health risks associated with severe mental illness

People living with mental illness are recognised as having reduced life expectancy in comparison with the general population and are seen to have worse levels of physical health, especially with respect to the cardiovascular system.^36^

Mental illnesses also complicate strategies for the prevention and management of medical illnesses. There are a variety of factors that are seen to affect the health inequalities that those with mental illness face, including poverty and social isolation, access to healthcare, genetics, treatment side-effects and lifestyle choices.^37^

## Physical health implications of HRT

HRT has faced a plethora of negative press coverage over the past 20 years. The Women's Health Institute randomised controlled trial^38^ is understood to have led to a decline in HRT prescriptions, as clinicians felt a significant level of uncertainty about the risk/benefit ratio of prescribing HRT. However, more reassuring outcomes following re-analysis of the data mean that the benefits of HRT can be more readily appreciated without concern about causing harm if appropriate HRT is prescribed.

By appropriate HRT, we mean body-identical HRT, which we know has no venous thromboembolic risk^39^ and causes no significant increase in breast cancer risk^40^ and is the gold-standard HRT that should be considered as first line therapy. In summary, body-identical HRT comprises transdermal oestradiol, in a patch, gel or spray, alongside micronised progesterone capsules for people who have a womb and require endometrial protection. An alternate means of providing endometrial protection is a Mirena coil. Further adjuncts include vaginal hormone preparations for genitourinary symptoms of menopause, which can indirectly affect psychological well-being,^41^ and transdermal testosterone, as a cream or gel, which is currently not licensed for use in women in the UK but can have a pivotal role in menopausal symptom management. Body-identical HRT is generally safe as it quite simply replaces the missing reproductive hormones, while improving symptoms as well as future health.^42^

November 2015 saw the National Institute for Health and Care Excellence publish guidance on the diagnosis and management of menopause.^43^ The British Menopause Society has issued a consensus statement on HRT,^44^ and there is also guidance from the International Menopause Society published in 2016.^45^ A simple prescribing guide summarising these guidelines and available evidence has been written by Newson Health Menopause Society in 2022.^46^

HRT can improve symptomatology of the perimenopause and menopause, which in itself can offer huge improvement in quality of life. There is also evidence that it can reduce risk of osteoporosis and cardiovascular disease, colon cancer, type II diabetes and cognitive decline.^35,47^

## HRT as a potential adjunct to psychotropic medication

HRT can be used to restore the declining levels of hormones in the perimenopause. Psychiatrists regularly assess and treat hypothyroidism, recognising it as an endocrinological condition with important psychological sequelae sometimes causing or at best exacerbating mental illness. The hormonal decline with perimenopause is more prevalent than hypothyroidism, and it is imperative that it is considered and managed alongside treatment plans for mental illness.

In new-onset low mood associated with the perimenopause, HRT (oestradiol and progestogen if needed) is the first line of treatment, and there is no evidence that antidepressants are beneficial.^43^ Current guidelines are to treat perimenopausal depression as any other depressive episode. However, it is known that peri- and postmenopausal people do not respond to selective serotonin reuptake inhibitors as effectively as other demographics,^16^ and rates of discontinuation due to adverse events are significant.^48^ There is limited evidence that desvenlafaxine is slightly efficacious in treating major depressive disorder in post- and perimenopausal patients, but other antidepressants have not been effectively scrutinised in this population.^1^ Oestradiol therapy has been shown to be beneficial in the treatment of perimenopausal depression both as a standalone agent and with even greater effect when combined with antidepressants.^1^

As discussed above, there is a strong correlation between vasomotor symptoms and anxiety symptoms. Venlafaxine is licensed for the treatment of vasomotor symptoms in the perimenopause, but HRT has been shown to be more effective.^49^

Throughout reproductive life, oestradiol is thought to raise the threshold of vulnerability to developing psychotic symptoms by being protective against schizophrenia;^9^ psychotic symptoms have been shown to be most severe in the premenstrual phase of the cycle when oestradiol levels are low.^50^ There have been case reports of new-onset perimenopausal psychosis failing to respond to antipsychotics but responding to HRT (oestradiol and progestogen) either as a sole agent^51^ or as an adjunct to antipsychotics.^52^

In premenopausal patients with chronic schizophrenia, transdermal oestradiol improves psychotic^53^ and depressive^54^ symptoms. A community study of postmenopausal women with psychotic illnesses found that those on HRT had fewer negative symptoms and needed lower doses of antipsychotics compared with women who had never received hormone therapy.^55^ A hypothesis with face validity is that using HRT to treat perimenopausal symptoms will optimise quality of life (e.g. improving sleep and reducing psychological distress and physical symptoms) and is likely to reduce the risk of relapse in schizophrenia.

There has been very little research into the role of HRT in the management of bipolar affective disorder through the perimenopause, but a small study found that perimenopausal women with a pre-existing diagnosis of bipolar affective disorder who did not use HRT had significant worsening of their symptoms of bipolar affective disorder compared with those who used HRT.^22^

## Hyperprolactinaemia with antipsychotics: an iatrogenic exacerbation of menopause?

**Fig. 1:**
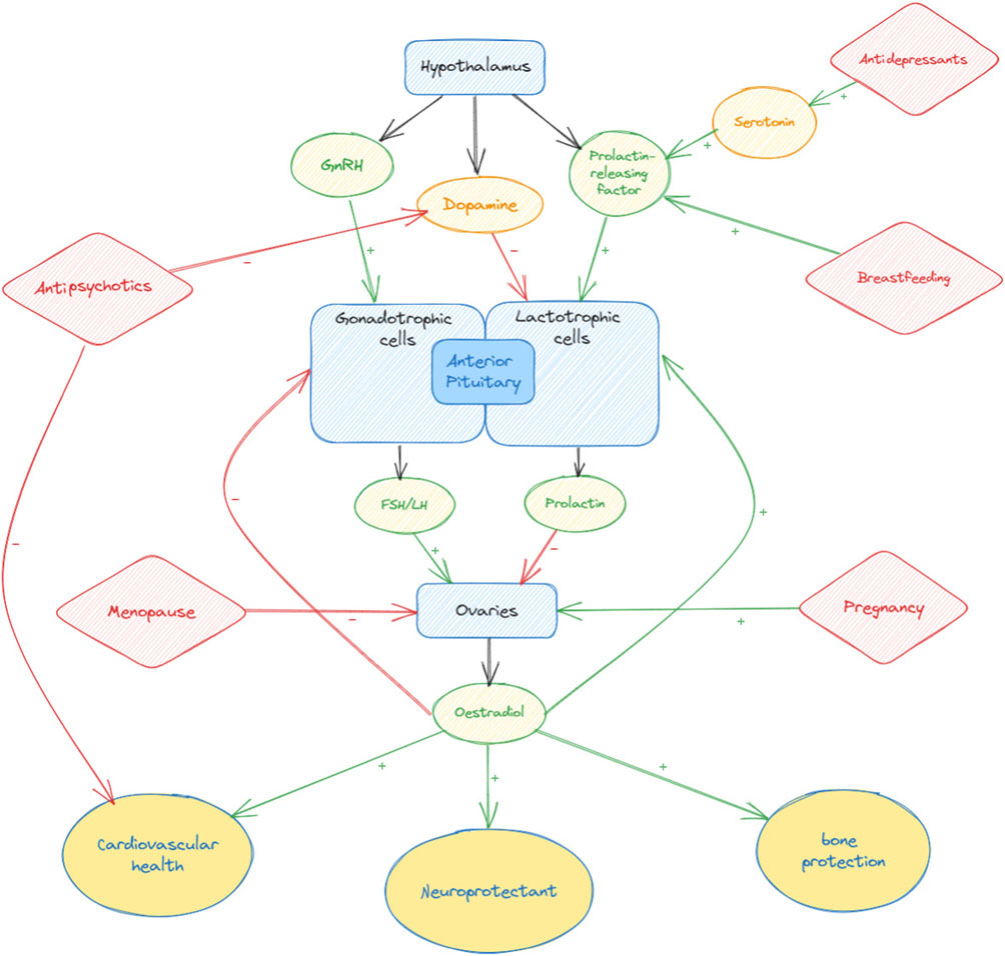
Interactions of psychotropic medication and hormonal events on the hypothalamopituitary–gonadal axis. The − symbol indicates inhibitory action; the + symbol indicates stimulation. FSH, follicle-stimulating hormone; GnRH, gonadotrophin-releasing hormone; LH, luteinising hormone.

Patients with chronic illnesses treated by antipsychotic medication (and to a lesser extent antidepressants) often have iatrogenic hyperprolactinaemia. Prolactin suppresses the hypothalamic secretion of gonadotrophin-releasing hormone, which leads to a suppression of follicle-stimulating hormone (FSH) and luteinising hormone and downstream suppression of sex hormones (oestradiol, progesterone and testosterone). This iatrogenic hypo-oestrogenic state may be exacerbated by the decline in oestradiol secondary to menopause (Fig. 1). It can also be hard to assess whether a patient with oligomenorrheoa is perimenopausal or not when prolactin is already high. Although measuring hormones to diagnose menopause is not usually recommended,^43^ in this scenario measuring FSH may be helpful, as raised FSH will give evidence that the patient may be perimenopausal as well as hyperprolactinaemic.

However, whatever the cause, a patient with new oligomenorrhoea will have low oestradiol levels and, as discussed below, this has negative implications for longer term physical health and so is worth treating, whether through adjustments to psychotropics or consideration of oestrogen as an adjunct. For this reason, it is particularly important to discuss menstrual cycles with patients on antipsychotics and/or approaching menopause and to work to optimise oestrogen levels in order to safeguard future physical and mental health.

## Conclusion

With perimenopausal patients with a history of mental illness, there is a danger of diagnostic overshadowing and new-onset perimenopausal symptoms being misdiagnosed as a relapse of a pre-existing mental illness. This can lead to delays in diagnosis and correct treatment. Women with schizophrenia are less likely to use HRT compared with women without psychiatric diagnosis.^56^ Just as we proactively investigate and treat physical comorbidities in patients with mental illness, we should also be proactive in discussion of the perimenopause and menopause, offering relevant lifestyle advice and HRT, if indicated.

When patients experience a relapse of their mental illness in the perimenopause, clinicians often prioritise the treatment of the mental illness with a view to maybe considering the perimenopausal symptoms when the acute mental illness is optimised; we would argue that this reductionist method is unhelpful, and that treating the perimenopause and mental illness in parallel is likely to lead to speedier recovery and better long-term outcomes. Discussion of symptoms through the prism of the perimenopause may be helpful for patients in understanding their experiences, and lifestyle advice may help with symptoms, which will have a positive impact on mental and physical health; the same is true for new presentations of perimenopause-related mental health difficulties. If oestrogen levels are balanced with HRT, patients are likely to respond more consistently to medication, perhaps requiring lower doses or less complex regimes and ultimately enjoying better physical and mental health outcomes.

## About the authors

**Sophie Behrman**, BA, BM, BCh, MRCPsych, is a consultant general adult psychiatrist working in a community mental health team in Oxford, employed by Oxford Health NHS Foundation Trust, UK. She is also an Honorary Clinical Research Fellow of the Oxford University Department of Psychiatry. **Clair Crockett**, BSc Hons, MBChB, DRCOG, MRCGP, DFSRH, is a general practitioner with a special interest in menopause employed by Newson Health Menopause and Wellbeing Centre, Stratford-upon-Avon, UK.
